# Causal Associations of Urate With Cardiovascular Risk Factors: Two-Sample Mendelian Randomization

**DOI:** 10.3389/fgene.2021.687279

**Published:** 2021-07-08

**Authors:** Thitiya Lukkunaprasit, Sasivimol Rattanasiri, Boonsong Ongphiphadhanakul, Gareth J. McKay, John Attia, Ammarin Thakkinstian

**Affiliations:** ^1^Department of Clinical Epidemiology and Biostatistics, Faculty of Medicine Ramathibodi Hospital, Mahidol University, Bangkok, Thailand; ^2^Department of Pharmacology, College of Pharmacy, Rangsit University, Pathum Thani, Thailand; ^3^Department of Medicine, Faculty of Medicine Ramathibodi Hospital, Mahidol University, Bangkok, Thailand; ^4^Centre for Public Health, School of Medicine, Dentistry and Biomedical Sciences, Queen’s University Belfast, Belfast, United Kingdom; ^5^Centre for Clinical Epidemiology and Biostatistics, School of Medicine and Public Health, Faculty of Health and Medicine, University of Newcastle, and Hunter Medical Research Institute, New Lambton, NSW, Australia

**Keywords:** cardiovascular risk factor, instrumental variable, Mendelian Randomization, urate, urate transporter gene

## Abstract

**Background:**

Mendelian Randomization (MR) studies show conflicting causal associations of genetically predicted serum urate with cardiovascular risk factors (i.e., hypertension, diabetes, lipid profile, and kidney function). This study aimed to robustly investigate a causal relationship between urate and cardiovascular risk factors considering single nucleotide polymorphisms (SNPs) as instrumental variables using two-sample MR and various sensitivity analyses.

**Methods:**

Data on SNP-urate associations were taken from the Global Urate Genetics Consortium and data on SNP-cardiovascular risk factor associations were taken from various consortia/UK Biobank. SNPs were selected by statistically and biologically driven approaches as instrumental variables. Various sensitivity analyses were performed using different MR methods including inverse variance weighted, MR-Egger, weighted median/mode, MR-PRESSO, and the contamination mixture method.

**Results:**

The statistically driven approach showed significant causal effects of urate on HDL-C and triglycerides using four of the six MR methods, i.e., every 1 mg/dl increase in genetically predicted urate was associated with 0.047 to 0.103 SD decrease in HDL-C and 0.034 to 0.207 SD increase in triglycerides. The biologically driven approach to selection of SNPs from *ABCG2, SLC2A9, SLC17A1, SLC22A11*, and *SLC22A12* showed consistent causal effects of urate on HDL-C from all methods with 0.038 to 0.057 SD decrease in HDL-C per 1 mg/dl increase of urate, and no evidence of horizontal pleiotropy was detected.

**Conclusion:**

Our study suggests a significant and robust causal effect of genetically predicted urate on HDL-C. This finding may explain a small proportion (7%) of the association between increased urate and cardiovascular disease but points to urate being a novel cardiac risk factor.

## Introduction

Cardiovascular disease (CVD) carries the greatest global disease burden (World Health Organization, 2017). It can be caused by various risk factors including serum urate (Feig et al., 2008). Previous meta-analyses have shown that elevated urate/hyperuricemia is associated not only with CVD (Kim et al., 2010) but also with CVD risk factors, i.e., hypertension (Grayson et al., 2011), type 2 diabetes mellitus (T2DM) (Kodama et al., 2009), metabolic syndrome (Yuan et al., 2015), and chronic kidney disease (CKD) (Li et al., 2014). Some studies also show that colchicine, a drug used for treatment of gout induced by urate crystal deposition, can reduce the risk of CVD (Tardif et al., 2019; Nidorf et al., 2020). However, it is unclear whether urate is truly causal for CVD, and if it is, whether this is through traditional risk factors or via a novel route.

Mendelian Randomization (MR) has been increasingly used to assess causal inference between gene(s), exposure/phenotype and clinical outcomes, considering that alleles are randomly allocated at meiosis and hence can be used as instrumental variables (IV) to test causation (Lawlor et al., 2008). Meiosis is similar to a randomized trial in that alleles are randomly assigned at conception, and hence unconfounded, except for ethnicity. The effect size of a single nucleotide polymorphism (SNP) is often too weak to serve as a robust IV, and thus genetic risk scores consisting of many SNPs are used to boost the effect size and increase the strength of the IV.

Two-sample MR is commonly used to assess causal association. It requires summary statistic data for SNP-phenotype and SNP-outcome associations that are derived from meta-analyses of genome-wide association studies (GWAS). Various statistical methods have been applied, e.g., inverse variance weighted (IVW) (Lawlor et al., 2008; Burgess et al., 2013), MR-Egger regression (Bowden et al., 2015), weighted median estimator (Bowden et al., 2016), weighted mode estimator (Hartwig et al., 2017), MR-PRESSO (Verbanck et al., 2018), and the contamination mixture method (Burgess et al., 2020b).

Several MR studies have been conducted to assess causal associations of genetic IVs related to urate and coronary heart disease (CHD) (Yang et al., 2010; Kleber et al., 2015; Keenan et al., 2016; White et al., 2016; Efstathiadou et al., 2019), hypertension (Kleber et al., 2015), body mass index (BMI) (White et al., 2016), fasting glucose (Yang et al., 2010; White et al., 2016), T2DM (Sluijs et al., 2015; Keenan et al., 2016; White et al., 2016), estimated glomerular filtration rate (eGFR) (Yang et al., 2010; Jordan et al., 2019), CKD (Yang et al., 2010; Jordan et al., 2019), and lipid profile (White et al., 2016). None suggested a causal association between urate and CHD (Yang et al., 2010; Kleber et al., 2015; Keenan et al., 2016; White et al., 2016), except one study (Efstathiadou et al., 2019) which suggested a modest causal association between urate and CHD, and one study (White et al., 2016) which found causal associations between 31 urate-SNPs and some CVD risk factors using the IVW method, which can be biased in the presence of pleiotropy (i.e., the IVs are associated with the outcome through pathways other than urate). Therefore, sensitivity analyses using other MR methods are recommended (Bowden et al., 2016; Burgess et al., 2020a). This study aimed to investigate a causal relationship between urate and CVD risk factors using genetic variants as the IVs, and two-sample MR with various sensitivity analyses to explore the robustness of the findings.

## Materials and Methods

**FIGURE 1:**
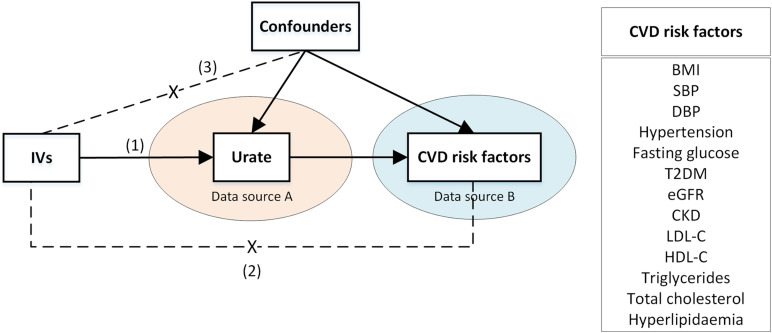
A two-sample Mendelian Randomization causal diagram with three instrumental variable assumptions. (1) IVs are associated with urate; (2) IVs have no direct effect on the CVD risk factors, except through urate; (3) IVs are not associated with confounders of urate -CVD risk factor association. Arrows represent associations. Dashed lines with a cross in the middle represent no associations. BMI, body mass index; CKD, chronic kidney disease; CVD, cardiovascular disease; DBP, diastolic blood pressure; eGFR, estimated glomerular filtration rate; HDL-C, high-density lipoprotein cholesterol; IV, instrumental variable; LDL-C, low-density lipoprotein cholesterol; SBP, systolic blood pressure; T2DM, type 2 diabetes mellitus.

We implemented two-sample MR complying with the STROBE-MR guidelines (Davey Smith et al., 2019) and the guidelines for performing MR (Burgess et al., 2020a). A MR causal-diagram was composed of genetic IVs, urate (as the exposure), and CVD risk factors (as outcomes, see Figure 1). Three IV assumptions were considered (Bowden et al., 2015); genetic IVs are strongly associated with urate; genetic IVs are associated with CVD risk factors only through urate; the associations between genetic IVs and urate and CVD risk factors are unconfounded.

### Data Sources

Summary data were retrieved from GWAS datasets via the MR-Base platform developed by the Medical Research Council Integrative Epidemiology Unit (MRC IEU); these datasets have already undergone the recommended quality control processes as previously described (Hemani et al., 2018). The data characteristics and the CVD risk factors considered are described in Table 1. Ethics approval and informed consent were previously obtained for all individual studies included. Data were retrieved for two pathways as follows:

**TABLE 1:** Characteristics of data sources.

Genetic associations	Consortium	Year	Sample size	Population	GWAS ID	Category	Unit of continuous traits
**SNP-exposure**							
SNP-urate	GUGC (Kottgen et al., 2013)	2013	110,347	European ancestry	ieu-a-1055	Continuous	mg/dl
**SNP-outcome (CVD risk factor)**							
SNP-SBP	MRC-IEU (Elsworth et al., 2017)	2018	436,419	European ancestry	ukb-b-20175	Continuous	SD
SNP-DBP	MRC-IEU (Elsworth et al., 2017)	2018	436,424	European ancestry	ukb-b-7992	Continuous	SD
SNP-hypertension	MRC-IEU (Elsworth et al., 2017)	2018	461,880 124,227 cases 337,653 controls	European ancestry	ukb-b-14177	Binary	NA
SNP-fasting glucose	MAGIC (Manning et al., 2012)	2012	58,074	European ancestry	ieu-a-773	Continuous	mmol/l
SNP-T2DM	DIAGRAM (Morris et al., 2012)	2012	69,033 12,171 cases 56,862 controls	European ancestry	ieu-a-26	Binary	NA
SNP-eGFR	CKDgen (Pattaro et al., 2016)	2015	133,814	Mainly European ancestry	ieu-a-1105	Continuous	Log ml/min/1.73 m^2^
SNP-CKD	CKDgen (Pattaro et al., 2016)	2015	117,165 12,385 cases 104,780 controls	Mainly European ancestry	ieu-a-1102	Binary	NA
SNP-BMI	GIANT (Locke et al., 2015)	2015	322,154	European ancestry	ieu-a-835	Continuous	SD (1 SD: 4.77 kg/m^2^)
SNP-LDL-C	GLGC (Willer et al., 2013)	2013	94,595	European ancestry	ebi-a-GCST002222	Continuous	SD (1 SD: 38.67 mg/dl)
SNP-HDL-C	GLGC (Willer et al., 2013)	2013	94,595	European ancestry	ebi-a-GCST002223	Continuous	SD mg/dl (1 SD: 15.51 mg/dl)
SNP-triglycerides	GLGC (Willer et al., 2013)	2013	94,595	European ancestry	ebi-a-GCST002216	Continuous	mg/dl (1 SD: 90.72 mg/dl)
SNP-total cholesterol	GLGC (Willer et al., 2013)	2013	94,595	European ancestry	ebi-a-GCST002221	Continuous	mg/dl (1 SD: 41.75 mg/dl)
SNP-hyperlipidemia	MRC-IEU (Elsworth et al., 2017)	2018	463,010 3439 cases 459,571 controls	European ancestry	ukb-b-17462	Binary	NA

*BMI, body mass index; CKD, chronic kidney disease; CKDgen, the Chronic Kidney Disease Genetics; CVD, cardiovascular disease; DBP, diastolic blood pressure; DIAGRAM, the DIAbetes Genetics Replication And Meta-analysis; eGFR, estimated glomerular filtration rate; GIANT, the Genetic Investigation of ANthropometric Traits; GLGC, the Global Lipids Genetics Consortium; GUGC, the Global Urate Genetics Consortium; GWAS, genome-wide association study; HDL-C, high-density lipoprotein cholesterol; ID, identification number; kg/m^2^, kilograms per square meter; LDL-C, low-density lipoprotein cholesterol; MAGIC, the Meta-Analyses of Glucose- and Insulin-related traits Consortium; NA, not applicable; mg/dl, milligrams per deciliter; ml/min/1.73 m^2^, milliliters per minute per 1.73 of square meter of body surface area; mmol/l, millimoles per liter; MRC-IEU, the MRC Integrative Epidemiology Unit; NA, not applicable; SBP, systolic blood pressure; SD, standard deviation; T2DM, type 2 diabetes mellitus.*

#### SNP → Urate Association

Single nucleotide polymorphism-urate associations were obtained from the Global Urate Genetics Consortium (GUGC), i.e., a meta-analysis of 48 GWAS in 110,347 Europeans (Kottgen et al., 2013). Mean age and percentage of males were 52.12 years and 45.15%, respectively. Mean (standard deviation; SD) urate ranged from 3.86 (0.92) to 6.10 (1.46) mg/dl; most studies used the uricase method for measuring urate. SNPs were selected as genetic IVs based on two approaches (Burgess et al., 2020a). First, a statistically driven approach was used to select SNPs that were highly associated with urate (*P*-value < 5 × 10^–8^) and in low linkage-disequilibrium with other SNPs (*r*^2^ < 0.001) within a clumping distance of 10,000 kb. Second, a biologically driven approach was used considering candidate genes *ABCG2, SLC2A9, SLC22A12, SLC22A11, SLC17A1*, and *SLC17A3* which encode urate transporters (Yang et al., 2010; Kottgen et al., 2013; Merriman, 2017); SNPs that were associated with urate (*P*-value < 5 × 10^–8^) and independent at *r*^2^ < 0.1 were selected. The summary data of the IVs on urate were extracted, F-statistic > 10 was used to evaluate if those SNPs were qualified IVs.

#### SNP → CVD Risk Factor Associations

Cardiovascular disease risk factors included blood pressure [i.e., systolic blood pressure (SBP)/diastolic blood pressure (DBP) and hypertension], T2DM and fasting glucose, renal outcomes (i.e., CKD and eGFR), lipid profile (i.e., LDL-C, HDL-C, triglycerides, total cholesterol, and hyperlipidemia), and BMI, see Table 1.

Summary data for SNP-BP associations (SBP/DBP and hypertension, defined as clinically diagnosed high blood pressure) were obtained from the MRC-IEU UK Biobank GWAS pipeline (Elsworth et al., 2017), see more details in Supplementary Note 1. Associations of SNP-SBP, SNP-DBP, and SNP-hypertension were obtained from >436,000 European participants.

Summary data for SNP-T2DM associations were obtained from the DIAbetes Genetics Replication And Meta-analysis (DIAGRAM) Consortium (Morris et al., 2012) and SNP-fasting glucose associations were from the Meta-Analyses of Glucose and Insulin-related traits Consortium (MAGIC) (Manning et al., 2012). The DIAGRAM Consortium data was extracted from the DIAGRAMv3 GWAS meta-analysis comprised of 12,171 T2DM cases and 56,862 controls of Europeans from 12 GWAS. T2DM was defined by original studies using several criteria including a fasting glucose ≥ 126 mg/dl, HbA1c ≥ 6.5%, self-report, medications used, etc. For MAGIC, data was included from >58,000 Europeans across 29 cohorts from the discovery stage.

Associations of SNP-CKD and SNP-eGFR were retrieved from the Chronic Kidney Disease Genetics (CKDGen) Consortium consisting of 43 studies for CKD (12,385 CKD cases and 104,780 controls) and 48 studies with 133,814 individuals for creatinine-based eGFR (eGFRcrea) (Pattaro et al., 2016). Participants were mainly of European descent. CKD was defined as eGFRcrea <60 ml/min/1.73m^2^. SNP-BMI summary data were retrieved from the Genetic Investigation of ANthropometric Traits (GIANT) Consortium (Locke et al., 2015), which included 125 studies from 322,154 European participants.

The SNPs-lipid profile data (i.e., LDL-C, HDL-C, triglycerides, and total cholesterol) were obtained from the Global Lipids Genetics Consortium (GLGC) (Willer et al., 2013), which pooled 23 GWAS from 94,595 Europeans. In addition, summary statistics for hyperlipidemia (3,439 cases and 459,571 controls), defined by ICD-10, were also retrieved from the MRC-IEU UK Biobank.

The effects of the genetic IVs on each CVD risk factor were extracted from source data as described above. Where specific SNP data was missing, a proxy SNP in linkage disequilibrium (*r*^2^ ≥ 0.8) was used instead (Hemani et al., 2018). All data were aligned to the Genome Reference Consortium Human Build 37 (GRCh37) and SNP identifiers were mapped to dbSNP build 144.

### Data Harmonization

Summary data for the SNP-urate and SNP-CVD risk factor associations [beta coefficient (β) and standard error (SE)] were retrieved and harmonized for comparisons of minor versus major (reference) alleles across databases. Palindromic SNPs (i.e., SNPs with A/T or G/C) with a minor allele frequency above 0.42 were excluded given the difficulty in identifying the reference strand when the minor allele frequency was close to 0.5 (Hemani et al., 2018).

### Statistical Analysis

The two-sample MR analyses were performed according to the guidelines (Burgess et al., 2020a). The main causal effect was estimated by a ratio of beta-coefficients of SNP-CVD risk factors to SNP-urate, and this was combined across all genetic IVs using the IVW method (Burgess et al., 2013) with a random-effect model. Five additional sensitivity analyses [i.e., MR-Egger (Bowden et al., 2015), weighted median (Bowden et al., 2016) and mode (Hartwig et al., 2017), MR-PRESSO (Verbanck et al., 2018), and the contamination mixture methods (Burgess et al., 2020b)] were performed to assess whether the causal estimates were robust to potential horizontal pleiotropy, see more details in Supplementary Note 2. Furthermore, leave-one-out analysis was conducted by removing one SNP at a time to observe the individual contributions to the IVW causal effects. In addition, bidirectional MR analysis was performed to identify the direction of any causal association. Steiger filtering was used to remove SNPs that were more correlated to the outcome than the exposure. Power calculations were performed, see Supplementary Tables 1A,B. R software version 3.6.3 was used for all analyses.

## Results

### Selection of Genetic IVs Based on the Statistically Driven Approach

There were 2,450,547 autosomal SNP-urate associations in the GUGC, of which 27 were significantly associated with urate with F statistics ranging from 35.39 to 1406.25 with corresponding *P*-values of 2.36 × 10^–8^ to 1.00 × 10^–200^; the proportion of phenotypic variance explained by each SNP (*R*^2^) ranged from 0.00032 to 0.01262, see Supplementary Table 2. Two palindromic SNPs (rs17632159 and rs6830367) were removed leaving 25 SNPs for SNP-urate and SNP-outcome associations, except for hyperlipidemia, where an additional two SNPs (rs7654258 and rs1825043) did not have outcome data, leaving 23 SNPs in total.

**FIGURE 2:**
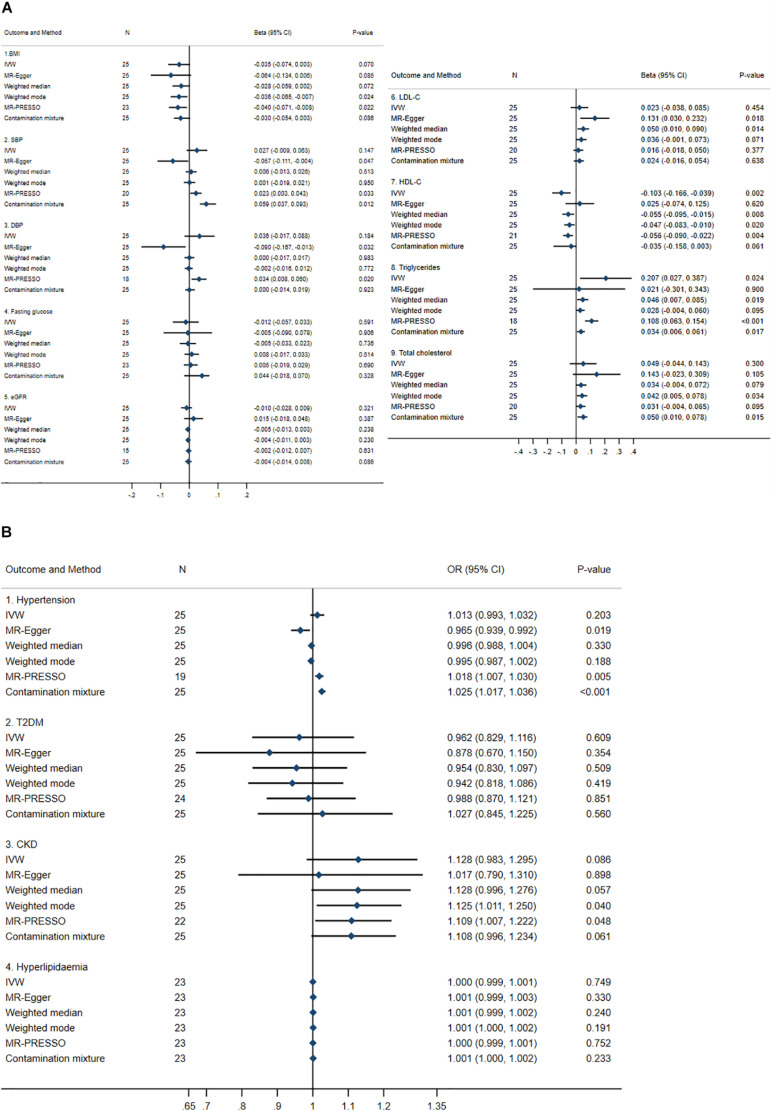
Summary causal effects of genetically predicted urate on cardiovascular risk factors by a statistically driven approach. **(A)** Continuous outcomes. **(B)** Binary outcomes. Refer to the unit of continuous outcomes in Table 1. Confidence interval (CI) from the contamination mixture method for LDL-C and T2DM contained multiple ranges of values (not shown in the figure). IVW, inverse variance weighted; MR-PRESSO, Mendelian Randomization Pleiotropy Residual Sum and Outlier; OR, odds ratio. For abbreviated outcomes, refer to abbreviation lists in Figure 1.

Mendelian Randomization results are described in Figures 2A,B. Between 15 and 25 SNPs were significantly associated with continuous outcomes through urate. Results from the six different MR methods were largely consistent for BMI, LDL-C, triglycerides, and total cholesterol with inconsistent causal effects for SBP, DBP, and HDL-C. For instance, two or three of the six MR methods showed significant causal effects of urate on SBP (i.e., MR-Egger, MR-PRESSO, and the contamination mixture method) and DBP (i.e., MR-Egger and MR-PRESSO) but the direction of the effects differed depending on the MR approach used. Four MR methods (i.e., IVW, weighted median, weighted mode, and MR-PRESSO) consistently showed significant causal effects of urate on HDL-C, i.e., for each 1 mg/dl increase in urate determined by genetic variants, there was a 0.047 to 0.103 SD decrease in HDL-C. In addition, the IVW method, weighted median estimator, MR-PRESSO, and the contamination mixture methods also demonstrated that for each 1 mg/dl increase in urate determined by genetic variants, there was a 0.034 to 0.207 SD increase in triglycerides, see Figure 2A. No causal effects of urate on fasting glucose and eGFR were observed.

For dichotomous outcomes, no causal effects of urate on T2DM or hyperlipidemia were identified but inconsistent causal effects on hypertension were observed. For hypertension, two of the six MR methods (i.e., MR-PRESSO and the contamination mixture method) detected causal risk effects with odds ratio (ORs) between 1.018 and 1.025, whereas MR-Egger detected an OR of 0.965. For CKD, two of the six MR methods (i.e., weighted-mode and MR-PRESSO) identified significant causal effects of urate with ORs of 1.109 and 1.125, respectively.

Heterogeneity was detected for all outcomes, see Supplementary Table 3. In addition, the pleiotropy test using the MR-Egger method identified significant pleiotropy with SBP, DBP, hypertension, LDL-C, and HDL-C, see Supplementary Table 3. It was noted that the contamination mixture method identified bimodal causal estimates of urate on LDL-C and T2DM, see Supplementary Table 4.

Additional leave-one-out plots were constructed, see Supplementary Figures 1A–M. Omitting a single SNP at a time did not significantly change the IVW causal estimates for any outcome, except BMI, SBP, CKD, and triglycerides, where the confidence intervals shifted from/to the null indicating non-robust causal estimates potentially due to outliers; scatter plots between genetically predicted urate and BMI, SBP, CKD, and triglycerides are shown in Supplementary Figures 2–5, respectively.

### Selection of Genetic IVs Based on the Biologically Driven Approach

Single nucleotide polymorphisms in six genes regulating urate transport were considered. Of these, 15 SNPs were significantly associated with urate (i.e., three SNPs in *ABCG2*, six SNPs in *SLC2A*9, four SNPs in *SLC17A1*, one SNP in *SLC22A11*, and one SNP in *SLC22A12*) with F-statistics of 49.00 to 2243.77 and corresponding *P*-values of 1.30 × 10^–10^ to 1.00 × 10^–200^ and *R*^2^ of 0.00045 to 0.01999, see Supplementary Table 5. Three SNPs in *SLC2A9* (rs10516194, rs13128385, and rs16891971) and rs10498730 in *SLC17A1* were removed due either to outcome data not being available or SNPs being palindromic; therefore, 11–14 SNPs remained in the analyses.

**FIGURE 3:**
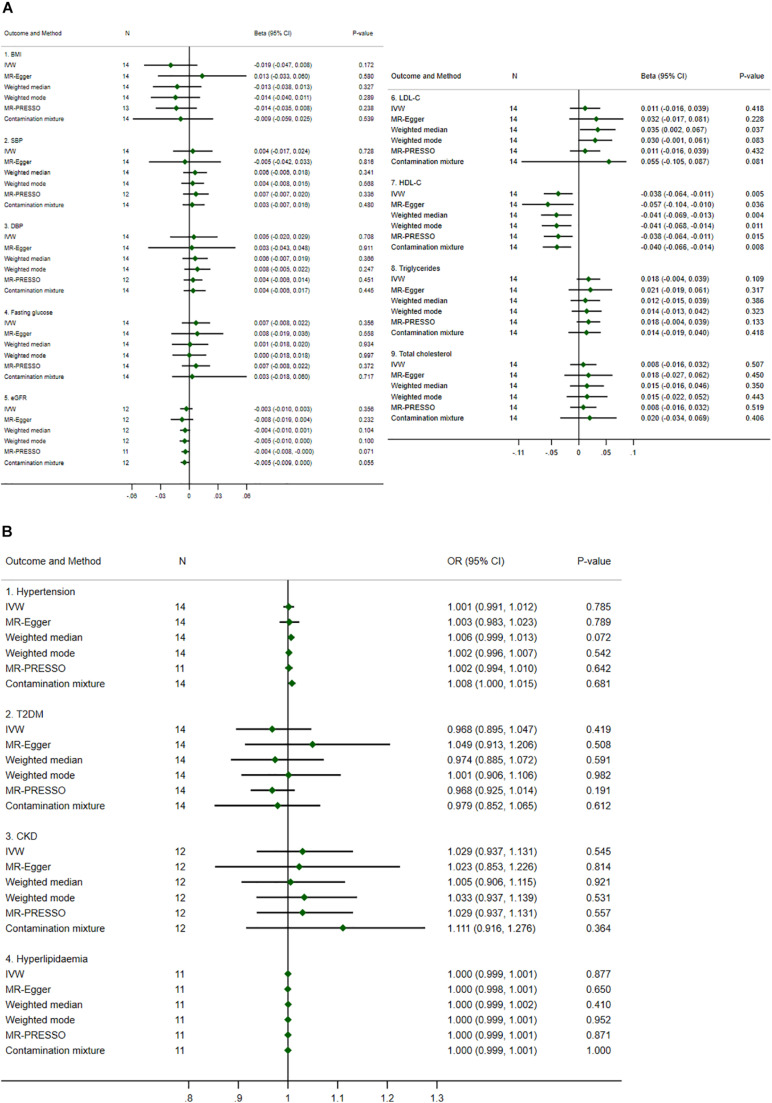
Summary causal effects of genetically predicted urate on cardiovascular risk factors by a biologically driven approach. **(A)** Continuous outcomes. **(B)** Binary outcomes. Refer to the unit of continuous outcomes in Table 1. Confidence interval (CI) from the contamination mixture method for hypertension contained multiple ranges of values (not shown in the figure). IVW, inverse variance weighted; MR-PRESSO, Mendelian Randomization Pleiotropy Residual Sum and Outlier; OR, odds ratio. For abbreviated outcomes, refer to abbreviation lists in Figure 1.

Mendelian Randomization results from the biologically driven approach are summarized (see Figures 3A,B), and were largely similar to those from the statistically driven approach but were more precise. For example, the causal estimates of urate on HDL-C were significant for all methods, i.e., for each 1 mg/dl increase in genetically predicted urate, HDL-C decreased by 0.038 to 0.057 SD. Sensitivity analysis to determine the validity of the genetic IVs using the contamination mixture method by varying SD (i.e., ψ) yielded consistent results, see Supplementary Table 6. Leave-one-out analysis also showed robust results for all outcomes, see Supplementary Figures 2A–M.

Heterogeneity was identified as borderline for HDL-C (*P*-value = 0.073 and 0.077 for IVW and MR-Egger methods, respectively), see Supplementary Table 7. The pleiotropy test assessed by the MR-Egger method was not significant for any causal estimates, see Supplementary Table 7. The confidence intervals for the causal estimates of urate on hypertension using the contamination mixture method did not converge and showed multiple ranges of values, see Supplementary Table 8.

We further performed bidirectional MR analysis to explore if genetically predicted HDL-C was causally associated with urate. A total 87 SNPs were highly associated with HDL-C with F-statistics ranging from 29.95 to 1749.13. Two palindromic SNPs were removed leaving 85 SNPs as genetic IVs. It was found that all MR methods except MR-Egger showed significant causal effects of HDL-C on urate, i.e., for each one SD increase in genetically predicted HDL-C, there was a 0.066 to 0.115 mg/dl decrease in urate, see Supplementary Note 3 and Supplementary Table 9.

## Discussion

We conducted two-sample MR assessing causal associations between genetically predicted urate and CVD risk factors using six analysis methods. Our results show robust causal associations between genetically predicted urate and HDL-C from the biologically driven approach. In addition, from the statistically driven approach, leave-one-out analyses suggested that rs1260326 in *GCKR* was the main genetic driver of urate on increased triglycerides and removing it resulted in a loss of significance. This finding is consistent with previous reported associations between rs1260326 and increased plasma triglycerides (Vaxillaire et al., 2008).

Six genes encoding urate transporters were considered in the biologically driven approach, i.e., *ABCG2, SLC17A1*, and *SLC17A3* involved in urate excretion whereas *SLC2A9, SLC22A12*, and *SLC22A11* regulated urate reabsorption in the renal proximal tubule (Merriman, 2017). The results showed significant causal estimates of these genetically predicted urate levels on decreased HDL-C, which were consistent across different MR methods. Sensitivity analyses using the leave-one-out method and varying the SD of invalid IVs using the contamination mixture method indicated robust results. In addition, the MR-Lasso and MR-RAPS methods also showed consistent significant results, see Supplementary Tables 10, 11. Furthermore, the causal estimates showed low heterogeneity and no evidence of pleiotropy, indicating valid genetic IVs. Our findings suggested that a genetically predicted increase of one unit in urate would decrease HDL-C by approximately 0.05 SD. A previous MR study (Allara et al., 2019) suggested that 1 SD increase in HDL-C would result in OR of CHD of 0.91. In other words, the OR for 1 SD decrease in HDL-C on CHD is about 1.10. The causal effects of 28 SNPs on CHD through urate was reported with an OR (estimated by MR-PRESSO) of 1.07 (Efstathiadou et al., 2019). If these causal effects are truly present, combining these figures using a mediation framework (Burgess et al., 2015), i.e., HDL-C mediates the effect of urate on CHD, leads to the estimate that 7% of the causal effect of genetically predicted urate on CHD is through HDL-C, see Supplementary Note 4. This would indicate that urate may be a novel risk factor for CVD and does not mediate its effect simply through other traditional risk factors. Inflammation plays a crucial role in CVD progression and emerging evidence suggests that colchicine, an anti-inflammatory medication used to treat gout, may also prove beneficial for the treatment of chronic coronary artery disease and the progression of atherosclerosis (Opstal et al., 2020).

Several studies previously conducted two-sample MR analysis with a statistically driven approach to assess causal relationships between urate and CVD risk factors. One study reported small causal effects of urate on HDL-C, triglycerides, SBP, and DBP, and no significant causal effects of urate on LDL-C, total cholesterol, fasting glucose, T2DM, and BMI (White et al., 2016). However, their results were based solely on the IVW method with no other MR methods to evaluate pleiotropy, so the causal effects may have been biased.

Another study identified inconsistent causal estimates of urate on SBP using different MR methods (Efstathiadou et al., 2019). Based on our statistically driven approach, only MR-PRESSO and the contamination mixture methods were able to identify the causal estimates of urate on SBP but these may have been biased as heterogeneity and pleiotropy were significantly present. In addition, a sensitivity analysis by varying the SD of invalid genetic IVs (ψ) in the contamination mixture method shifted the lower confidence interval toward the null, questioning the validity of the instruments. This method also detected multiple ranges across the confidence intervals of the causal estimates on both SBP and hypertension, implicating more than one biological mechanism linking urate and SBP or hypertension (data not shown). Our leave-one-out analyses also showed that rs2231142 in *ABCG2* was influential on the effect of urate on SBP.

Another recent study revealed no evidence of causal effects of urate on either eGFR or CKD from different MR methods (Jordan et al., 2019), which was similar to our statistically driven approach by the contamination mixture method. Furthermore, we found no causal effects of urate on fasting glucose and T2DM in line with other MR studies using individual level data and different MR approaches (Kleber et al., 2015; Sluijs et al., 2015).

Our findings suggest that urate may be causally related to HDL-C levels. This supports a previous meta-analysis demonstrating a significant inverse relationship between urate and HDL-C (Chen et al., 2020b), although longitudinal studies reported inconsistent findings (Gonçalves et al., 2012; Babio et al., 2015). One small study observed significantly higher HDL-C after 3 months of allopurinol administration, a xanthine oxidase inhibitor used for hyperuricemia treatment (Ziga and Becic, 2013). However, randomized controlled trials did not find a significant change in HDL-C levels in CKD and CVD patients receiving xanthine oxidase inhibitors (Bowden et al., 2013; Nakagomi et al., 2015). Nevertheless, there are a number of biological mechanisms suggesting that urate may reduce HDL-C. It has been shown that higher fructose consumption leads to increased circulating urate and metabolic syndrome, and, by reducing urate through allopurinol, the derangements in the metabolic features can be alleviated (Nakagawa et al., 2006). Another potential mechanism may be mediated through fibroblast growth factor 21 (FGF21), a metabolic regulator that demonstrates glucose and lipid-lowering effects in various animal models (Xie and Leung, 2017). In diabetic monkeys, the administration of recombinant FGF21 improves the blood lipid profile including increasing HDL-C in a dose-dependent manner (Kharitonenkov et al., 2007). Recently, miR-149-5p, which targets FGF21, was shown to be significantly up-regulated in uric acid-stimulated hepatocytes, leading to aggregated uric acid-induced triglyceride accumulation (Chen et al., 2020a). Taken together, it is probable that urate is causally related to reduced HDL-C. Surprisingly, bidirectional MR indicated that a reverse causal association between genetically predicted HDL-C and urate may also be present; the mechanism for this may be that an increase in apolipoprotein-A1 (the main component of HDL-C) is associated with high eGFR (Goek et al., 2012), which consequently increases uric acid excretion through urine, thus lowering serum urate level as previously proposed (Peng et al., 2015).

Our study has several strengths. Two-sample MR analysis increases study power through the inclusion of larger sample sizes from GWAS consortia. MR is also less influenced by potential confounding due to the random allocation of alleles at conception. The genetic IVs were carefully selected by the statistically and biologically driven hypotheses to strengthen the robustness of the results. Our findings favor the biologically driven approach, where SNPs were selected from genes with biological plausibility, leading to lower heterogeneity and pleiotropy than the statistically driven approach. The data sources used were mostly selected from populations of European ancestry, therefore, population stratification is much less likely. These data also originate from public sources, so our approach is transparent and reproducible. We have comprehensively performed six different MR methods to account for pleiotropy and observed robust results, including the contamination mixture method, a novel MR method that has been proposed to better address the issue of invalid instruments (Burgess et al., 2020b). The urate and HDL-C causal relationship was supported by the robust identification of genetic variants in urate transporters.

### Limitations

Some limitations could be not avoided. We used two-sample MR methods, where datasets for SNP-urate and SNP-outcomes were derived from different individuals. A degree of overlap in data sources for SNP-urate (i.e., GUGC) and the outcome consortia (i.e., CKDgen, DIAGRAM, MAGIC, GIANT, and GLGC) might also affect causal estimates. The results of LD score regression analysis provided in LD Hub (Bulik-Sullivan et al., 2015; Zheng et al., 2017) indicate that the intercept of genetic correlations between urate and HDL-C was 1.057, indicating some degree of sample overlap. Lastly, some outcome data (e.g., T2DM and hypertension) were defined slightly differently across GWAS, making absolute standardization impossible based on summary-aggregated data.

## Conclusion

Our data indicates a significant causal effect of genetically predicted urate on HDL-C and supports recent randomized controlled trial data suggesting that colchicine, a commonly prescribed drug for the treatment of gout, reduces the risk of CVD. Furthermore, our results suggest that only a small component (7%) of this association may be mediated by known risk factors, especially HDL, and that the majority of this effect is likely mediated by other, novel routes.

## Data Availability Statement

Publicly available datasets were analyzed in this study. This data can be found here: https://www.mrbase.org.

## Ethics Statement

This study involving human participants was reviewed and approved by Institutional Review Board of the Faculty of Medicine, Ramathibodi Hospital, Mahidol University. Written informed consent for participation was not required for this study in accordance with the national legislation and the institutional requirements.

## Author Contributions

TL designed the study, performed data acquisition, analysis, interpretation, and drafted the manuscript. SR performed data acquisition and analysis. BO, GJM, and JA performed data interpretation. AT designed the study and performed data interpretation. All authors read and approved the final manuscript.

## Conflict of Interest

The authors declare that the research was conducted in the absence of any commercial or financial relationships that could be construed as a potential conflict of interest.
