# Supplementary material for: Causal Associations of Urate With Cardiovascular Risk Factors: Two-Sample Mendelian Randomization
Source: Front Genet. 2021 Jul 8;12:687279. doi: 10.3389/fgene.2021.687279 (PMC8297413; doi:10.3389/fgene.2021.687279)
Supplement: Supplementary file 1 [file Data_Sheet_1.docx]

**Supplementary materials**

[Supplementary Note 1. MRC-IEU UK Biobank GWAS pipeline 3](#_Toc74952683)

[Supplementary Note 2. Statistical analysis 4](#_Toc74952684)

[Supplementary Note 3. Bidirectional MR analysis 5](#_Toc74952685)

[Supplementary Note 4. Calculation of the causal effect of urate on coronary heart disease (CHD) mediated through HDL-C 6](#_Toc74952686)

[Supplementary Table 1. Power calculation for two-sample Mendelian randomization 7](#_Toc74952687)

[Supplementary Table 2. Associations between selected SNPs and urate based on statistically driven approach 9](#_Toc74952688)

[Supplementary Table 3. Heterogeneity and pleiotropy test results using genetic instrumental variables based on statistically driven approach 10](#_Toc74952689)

[Supplementary Table 4. Causal associations of urate with LDL-C and T2DM estimated by the contamination-mixture method based on statistically driven approach 11](#_Toc74952690)

[Supplementary Table 5. Associations between selected SNPs and urate based on biologically driven approach 12](#_Toc74952691)

[Supplementary Table 6. Causal associations of urate with HDL-C estimated by the contamination mixture method at different values of standard deviation parameter (*ψ)* based on biologically driven approach 13](#_Toc74952692)

[Supplementary Table 7. Heterogeneity and pleiotropy test results using genetic instrumental variables based on biologically driven approach 14](#_Toc74952693)

[Supplementary Table 8. Causal associations of urate with hypertension from the contamination mixture method based on biologically driven approach 15](#_Toc74952694)

[Supplementary Table 9. Causal associations of HDL-C with urate: bidirectional MR 16](#_Toc74952695)

[Supplementary Table 10. Causal associations of urate with CVD risk factors estimated by MR-Lasso and MR-RAPS based on statistically driven approach 17](#_Toc74952696)

[Supplementary Table 11. Causal associations of urate with CVD risk factors estimated by MR-Lasso and MR-RAPS based on biologically driven approach 18](#_Toc74952697)

[Supplementary Figure 1. Leave-one-out plots of associations between urate and cardiovascular risk factors based on statistically driven approach. 19](#_Toc74952698)

[A) Body mass index (BMI) 19](#_Toc74952699)

[B) Systolic blood pressure (SBP) 20](#_Toc74952700)

[C) Diastolic blood pressure (DBP) 21](#_Toc74952701)

[D) Hypertension 22](#_Toc74952702)

[E) Fasting glucose 23](#_Toc74952703)

[F) Type 2 diabetes mellitus (T2DM) 24](#_Toc74952704)

[G) Estimated glomerular filtration rate (eGFR) 25](#_Toc74952705)

[H) Chronic kidney disease (CKD) 26](#_Toc74952706)

[I) Low-density lipoprotein cholesterol (LDL-C) 27](#_Toc74952707)

[J) High-density lipoprotein cholesterol (HDL-C) 28](#_Toc74952708)

[K) Triglycerides 29](#_Toc74952709)

[L) Total cholesterol 30](#_Toc74952710)

[M) Hyperlipidaemia 31](#_Toc74952711)

[Supplementary Figure 2. Scatter plots of associations between urate and BMI 32](#_Toc74952712)

[Supplementary Figure 3. Scatter plots of associations between urate and SBP 33](#_Toc74952713)

[Supplementary Figure 4. Scatter plots of associations between urate and CKD 34](#_Toc74952714)

[Supplementary Figure 5. Scatter plots of associations between urate and triglycerides 35](#_Toc74952715)

[Supplementary Figure 6. Leave-one-out plots of associations between urate and cardiovascular risk factors based on biologically driven approach 36](#_Toc74952716)

[A) Body mass index (BMI) 36](#_Toc74952717)

[B) Systolic blood pressure (SBP) 37](#_Toc74952718)

[C) Diastolic blood pressure (DBP) 38](#_Toc74952719)

[D) Hypertension 39](#_Toc74952720)

[E) Fasting glucose 40](#_Toc74952721)

[F) Type 2 diabetes mellitus (T2DM) 41](#_Toc74952722)

[G) Estimated glomerular filtration rate (eGFR) 42](#_Toc74952723)

[H) Chronic kidney disease (CKD) 43](#_Toc74952724)

[I) Low-density lipoprotein cholesterol (LDL-C) 44](#_Toc74952725)

[J) High-density lipoprotein cholesterol (HDL-C) 45](#_Toc74952726)

[K) Triglycerides 46](#_Toc74952727)

[L) Total cholesterol 47](#_Toc74952728)

[M) Hyperlipidaemia 48](#_Toc74952729)

[References 49](#_Toc74952730)

# Supplementary Note 1. MRC-IEU UK Biobank GWAS pipeline

The UK Biobank is a population-based health research resource consisting of approximately 500,000 people, aged between 38 years and 73 years, who were recruited between the years 2006 and 2010 from across the UK (Allen et al., 2014). As the UK Biobank focused on identifying determinants of human diseases in middle-aged and older individuals, participants provided a range of information (such as demographics, health status, lifestyle measures, cognitive testing, personality self-report, and physical and mental health measures) via questionnaires and interviews; anthropometric measures, BP readings and samples of blood, urine and saliva were collected (data available at www.ukbiobank.ac.uk). The population was restricted to those of European ancestry. A full description of the study design, participants and quality control (QC) methods have been described previously (Collins, 2012). UK Biobank received ethical approval from the Research Ethics Committee (REC reference for UK Biobank is 11/NW/0382). The quality control filtering was conducted by R.Mitchell, G.Hemani, T.Dudding, L.Paternoster as described in the published protocol (doi:10.5523/bris.3074krb6t2frj29yh2b03x3wxj). The MRC IEU UK Biobank GWAS pipeline was developed by B.Elsworth, R.Mitchell, C.Raistrick, L.Paternoster, G.Hemani, T.Gaunt (doi: 10.5523/bris.2fahpksont1zi26xosyamqo8rr).

# Supplementary Note 2. Statistical analysis

The two-sample MR analyses were performed in accordance with recently published guidelines (Burgess et al., 2020). The main causal effect was estimated by a ratio of beta-coefficients of SNP-CVD risk factors to SNP-urate and combined across all genetic IVs using the IVW method (Burgess et al., 2013) with multiplicative random-effect model. The IVW may not be valid if horizontal pleiotropy exists. Therefore, sensitivity analyses were performed using five additional methods to assess whether the causal estimates were robust to potential horizontal pleiotropy. The MR-Egger method (Bowden et al., 2015), similar to the Egger regression used in general meta-analysis, was applied by weighted regression of SNP-outcome effects on SNP-phenotype effects. The intercept of the regression line represents the average pleiotropic effect and the slope is the causal estimate corrected for pleiotropy under the assumption that the SNP-urate association and the pleotropic effects are not correlated; this is known as the instrument strength independent of direct effects (InSIDE) assumption.

Weighted median (Bowden et al., 2016) and mode (Hartwig et al., 2017) estimators were also applied. The former estimates a causal effect by a median of the weighted empirical distribution function of ordered ratios of beta coefficients of SNP-CVD risk factors to SNP-urate which provides a reliable estimate when at least 50% of the instrumental variables are valid. The weighted mode estimator uses the mode of the weighted empirical density function of ratio effects which provides a reliable estimate when the largest group of genetic instrumental variables are valid with similar causal estimates (plurality assumption).

The MR-PRESSO (Mendelian Randomization Pleiotropy Residual Sum and Outlier) method (Verbanck et al., 2018) was used to assess pleiotropy by comparing the observed and expected residual sum squares of all genetic IVs from the global test. The causal effect by the IVW method is estimated after removal of genetic outliers showing pleiotropy (the outlier test) and the causal effects before and after removing outliers were compared using the distortion test.

The contamination mixture method (Burgess et al., 2020) is based on constructing likelihood functions of two-component mixture distributions for each variant as valid and invalid IVs. If the likelihood of a valid instrument is higher, then the variant is considered a valid instrument. Conversely, if the likelihood as an invalid variant is higher, that variant is considered an invalid instrument. A sensitivity analysis was performed by varying the SD of the invalid estimands (*ψ*) initially at 1.5 times the SD of the ratio estimates for the valid instrumental variables. This method also identifies subgroups of instrumental variables with similar causal estimates and implies multiple biological mechanisms when the likelihood function is multimodal.

Leave-one-out analysis was also conducted following the removal of a SNP at a time to observe the individual contributions to the IVW causal effects. Power calculations were performed, see Supplementary Table 1A-B. R software version 3.6.3 was used for all analyses. TwoSampleMR package (version 0.5.1) was used for the IVW method, MR-Egger method, and weighted median/mode. MR-PRESSO and Mendelian Randomization packages were used for MR-PRESSO and the contamination mixture method, respectively.

# Supplementary Note 3. Bidirectional MR analysis

We further performed bidirectional MR analysis to explore if genetically predicted HDL-C is causally associated with urate. A total 87 SNPs were highly associated with HDL-C with F-statistics ranging from 29.95 to 1749.13. Two palindromic SNPs were removed leaving 85 SNPs qualified as genetic IVs. It was found that all MR methods except MR-Egger showed significant causal effects of HDL-C on urate, i.e., for each one SD increase in genetically predicted HDL-C, there was a 0.066 to 0.115 mg/dl decrease in urate. Heterogeneity and pleiotropy were detected (Cochran’s Q test P-value <0.01, pleiotropy test P-value = 0.016). After removing SNPs that were significantly correlated with the outcome more than the exposure as suggested by Steiger filtering, the causal estimates were still similar for the direction of HDL-C to urate (0.064 to 0.106 mg/dl decrease in urate, rs3741414 removed) and also still similar for the direction of urate to HDL-C (0.043 to 0.081 SD decrease in HDL-C, rs1178977 and rs653178 removed). For the causal effect of urate on HDL-C based on the biologically driven approach, no any SNP was correlated to HDL-C more than urate from Steiger filtering, and therefore none was removed.

# Supplementary Note 4. Calculation of the causal effect of urate on coronary heart disease (CHD) mediated through HDL-C

| **Urate → CHD** | | | |
| --- | --- | --- | --- |
| **Author** | **Method** | **OR (95% CI)** | **Note** |
| (Efstathiadou et al., 2019) | Fixed effects IVW | 1.08 (1.02–1.14) | - 28 urate SNPs at p<5x10^-8^  - OR per 1 mg/dl increase of urate |
|  | Weighted median | 1.05 (0.99–1.11) |  |
|  | MR-Egger | 1.02 (0.94–1.12) |  |
|  | MR-PRESSO (outlier corrected) | 1.07 (1.03–1.12) |  |
|  | Multivariable MR  (adjusted for BMI) | 1.07 (1.01–1.14) |  |
|  | MR-Egger | 1.05 (0.92-1.20) |  |
|  | Multivariable MR  (adjusted for SBP, DBP, TG, HDL-C) | 1.10 (1.00-1.22) |  |
| **HDL-C → CHD** | | | |
| **Author** | **Method** | **OR (95% CI)** | **Note** |
| (Allara et al., 2019) | IVW | 0.82 (0.74-0.92) | - HDL-C SNPs at p<5x10^-8^  - OR per 1 SD increase of HDL-C |
|  | MR-Egger | 0.99 (0.83-1.19) |  |
|  | Weighted median | 0.85 (0.78-0.92) |  |
|  | Multivariable MR | 0.91 (0.83–1.00)  P value = 0.043 |  |

**
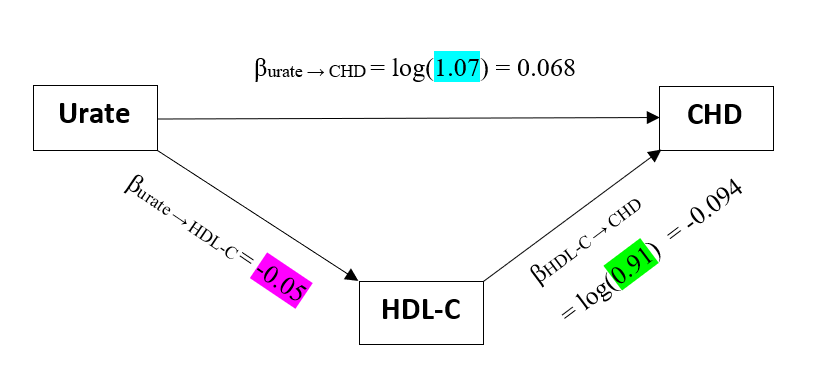
**

**Calculation of % contribution** **of urate → HDL-C → CHD**

Our 2-sample MR study shows that 1 mg/dl increase of urate is significantly associated with approximately 0.05 SD unit decrease of HDL-C.

% Contribution of urate → HDL-C → CHD $=\frac{\beta_{urate \to HDL-C} \times\beta_{HDL-C \to CHD}}{\beta_{urate \to CHD}} \times100$

$=\frac{\left( -0.05 \right)\times(-0.094)}{0.068}\times100$

$=6.91\%$

# Supplementary Table 1. Power calculation for two-sample Mendelian randomization

1. Continuous outcomes

| Outcome | N | R^2^ of the instrument (Kottgen et al., 2013) | Causal effect | References | Power |
| --- | --- | --- | --- | --- | --- |
| BMI | 322,154 | 0.07 | 0.376 | (Yu et al., 2016) | 100% |
| SBP | 436,419 | 0.07 | 0.122 | (Yu et al., 2016) | 100% |
| DBP | 436,424 | 0.07 | 0.195 | (Yu et al., 2016) | 100% |
| Fasting glucose | 58,074 | 0.07 | 0.057 | (Yu et al., 2016) | 95.3% |
| eGFR | 133,814 | 0.07 | -0.231 | (Yu et al., 2016) | 100% |
| LDL-C | 94,595 | 0.07 | 0.141 | (Yu et al., 2016) | 100% |
| HDL-C | 94,595 | 0.07 | -0.279 | (Yu et al., 2016) | 100% |
| Triglycerides | 94,595 | 0.07 | 0.279 | (Yu et al., 2016) | 100% |
| Total cholesterol | 94,595 | 0.07 | 0.071 | (Kim et al., 2019) | 100% |

Power calculation for continuous outcomes from online power calculator at <https://sb452.shinyapps.io/power/>. Causal effect is change in outcome in SD units per SD change in exposure, i.e., Pearson’s correlation (*r*) according to the equation $b=r\frac{S_{y}}{S_{x}}$ ,where *b* is the slope from linear regression equation and *S_y_* and S*_x_* are standard deviation of outcome (*y*) and exposure (*x*), respectively. Abbreviations: BMI, body mass index; DBP, diastolic blood pressure; eGFR, estimated glomerular filtration rate; HDL-C, high-density lipoprotein cholesterol; LDL-C, low-density lipoprotein cholesterol; SBP, systolic blood pressure.

1. Binary outcomes

| Outcome | N | R^2^ of the instrument  (Kottgen et al., 2013) | Proportion of cases | OR | References | Power |
| --- | --- | --- | --- | --- | --- | --- |
| Hypertension | 461,880 | 0.07 | 0.27 | 1.07 | (Grayson et al., 2011) | 100% |
|  |  |  |  | 1.26 | (Grayson et al., 2011) | 100% |
| CKD | 117,165 | 0.07 | 0.11 | 1.01 | (Weiner et al., 2008) | 6% (11,478,302 samples required at 80% power) |
|  |  |  |  | 1.14 | (Weiner et al., 2008) | 97% |
| T2DM | 69,033 | 0.07 | 0.18 | 1.28 | (Xu et al., 2016) | 100% |
|  |  |  |  | 1.72 | (Xu et al., 2016) | 100% |
| Hyperlipidaemia | 463,010 | 0.07 | 0.007 | 1.17 | (Chen et al., 2020) | 72% |
|  |  |  |  | 1.37 | (Chen et al., 2020) | 100% |

Power calculation for binary outcomes from online power calculator at <https://shiny.cnsgenomics.com/mRnd/>. OR values are based on the lower and upper limit of 95% confidence interval from the references.

Abbreviations: CKD, chronic kidney disease; OR, odds ratio; T2DM, type 2 diabetes mellitus.

# Supplementary Table 2. Associations between selected SNPs and urate based on statistically driven approach

| SNP | Chr. | Gene | Effect allele | Other allele | SNP-urate association | | | R^2^ | F-statistic |
| --- | --- | --- | --- | --- | --- | --- | --- | --- | --- |
|  |  |  |  |  | Beta coefficient | SE | P-value |  |  |
| rs11264341 | 1 | *TRIM46* | T | C | -0.048 | 0.006 | 1.04E-14 | 0.00063 | 66.19 |
| rs1471633 | 1 | *PDZK1* | C | A | -0.061 | 0.005 | 1.40E-26 | 0.00117 | 127.61 |
| rs1260326 | 2 | *GCKR* | C | T | -0.077 | 0.006 | 1.31E-40 | 0.00178 | 196.00 |
| rs17050272 | 2 | *INHBB* | A | G | 0.037 | 0.006 | 9.36E-09 | 0.00036 | 36.79 |
| rs2307394 | 2 | *ORC4L* | C | T | 0.035 | 0.006 | 7.26E-09 | 0.00034 | 37.70 |
| rs6770152 | 3 | *SFMBT1* | T | G | -0.048 | 0.006 | 2.66E-16 | 0.00067 | 73.47 |
| rs11722228 | 4 | *SLC2A9* | T | C | 0.210 | 0.006 | 1.00E-200 | 0.01262 | 1406.25 |
| rs1825043 | 4 | Intergenic region between *RPS3AP19* and LOC100421806 | A | G | 0.100 | 0.013 | 6.67E-14 | 0.00054 | 59.17 |
| rs2231142 | 4 | *ABCG2* | T | G | 0.220 | 0.009 | 4.44E-116 | 0.00528 | 584.47 |
| rs6830367 | 4 | *CLNK* | C | G | 0.051 | 0.007 | 2.35E-11 | 0.00046 | 50.17 |
| rs7654258 | 4 | *LOC107986260* | T | C | -0.099 | 0.010 | 1.09E-19 | 0.00093 | 98.01 |
| rs17632159 | 5 | *TMEM171* | C | G | -0.038 | 0.006 | 2.00E-09 | 0.00036 | 38.81 |
| rs1165151 | 6 | *SLC17A1* | G | T | 0.092 | 0.005 | 4.52E-60 | 0.00263 | 290.26 |
| rs675209 | 6 | *RREB1* | C | T | -0.063 | 0.006 | 1.38E-21 | 0.00094 | 103.25 |
| rs729761 | 6 | *VEGFA* | G | T | 0.046 | 0.006 | 3.05E-12 | 0.00049 | 53.31 |
| rs1178977 | 7 | *BAZ1B* | G | A | -0.050 | 0.007 | 6.68E-12 | 0.00048 | 52.51 |
| rs2941484 | 8 | *HNF4G* | T | C | 0.049 | 0.006 | 3.91E-17 | 0.00072 | 79.37 |
| rs10761587 | 10 | *A1CF* | C | T | -0.062 | 0.009 | 9.58E-11 | 0.00043 | 46.42 |
| rs1171614 | 10 | *SLC16A9* | C | T | 0.074 | 0.007 | 6.48E-23 | 0.00105 | 108.63 |
| rs2078267 | 11 | *SLC22A11* | T | C | -0.078 | 0.006 | 8.73E-36 | 0.00178 | 174.78 |
| rs642803 | 11 | *OVAL1* | T | C | -0.043 | 0.005 | 4.51E-14 | 0.00058 | 63.41 |
| rs3741414 | 12 | *INHBC* | T | C | -0.071 | 0.007 | 9.79E-22 | 0.00100 | 102.88 |
| rs653178 | 12 | *ATXN2* | T | C | -0.036 | 0.005 | 2.45E-10 | 0.00040 | 44.44 |
| rs1394125 | 15 | *UBE2Q2* | A | G | 0.043 | 0.006 | 9.78E-11 | 0.00044 | 46.59 |
| rs6598541 | 15 | *IGF1R* | G | A | -0.044 | 0.006 | 5.20E-13 | 0.00055 | 59.59 |
| rs7193778 | 16 | *NFAT5* | T | C | -0.047 | 0.008 | 2.36E-08 | 0.00032 | 35.39 |
| rs7224610 | 17 | *HLF* | A | C | -0.038 | 0.006 | 4.74E-11 | 0.00043 | 47.74 |

Abbreviations: Chr., chromosome; SE, standard error; SNP, single nucleotide polymorphism.

# Supplementary Table 3. Heterogeneity and pleiotropy test results using genetic instrumental variables based on statistically driven approach

| Continuous outcome | Method | Beta | 95% CI | P-value | |
| --- | --- | --- | --- | --- | --- |
|  |  |  |  | Cochran’s Q test | Pleiotropy test |
| BMI | IVW | -0.035 | -0.074, 0.003 | <0.001 |  |
|  | MR-Egger | -0.064 | -0.134, 0.006 | <0.001 | 0.346 |
| SBP | IVW | 0.027 | -0.009, 0.063 | <0.001 |  |
|  | MR-Egger | -0.057 | -0.111, -0.004 | <0.001 | 0.001 |
| DBP | IVW | 0.036 | -0.017, 0.088 | <0.001 |  |
|  | MR-Egger | -0.090 | -0.167, -0.013 | <0.001 | 0.001 |
| Fasting glucose | IVW | -0.012 | -0.057, 0.033 | <0.001 |  |
|  | MR-Egger | -0.005 | -0.090, 0.079 | <0.001 | 0.844 |
| eGFR | IVW | -0.010 | -0.028, 0.009 | <0.001 |  |
|  | MR-Egger | 0.015 | -0.018, 0.048 | <0.001 | 0.095 |
| LDL-C | IVW | 0.023 | -0.038, 0.085 | <0.001 |  |
|  | MR-Egger | 0.131 | 0.030, 0.232 | <0.001 | 0.020 |
| HDL-C | IVW | -0.103 | -0.166, -0.039 | <0.001 |  |
|  | MR-Egger | 0.025 | -0.074, 0.125 | <0.001 | 0.006 |
| Triglycerides | IVW | 0.207 | 0.027, 0.387 | <0.001 |  |
|  | MR-Egger | 0.021 | -0.301, 0.343 | <0.001 | 0.187 |
| Total cholesterol | IVW | 0.049 | -0.044, 0.143 | <0.001 |  |
|  | MR-Egger | 0.143 | -0.023, 0.309 | <0.001 | 0.197 |
| Binary outcome | Method | OR | 95% CI | P-value | |
|  |  |  |  | Cochran’s Q test | Pleiotropy test |
| Hypertension | IVW | 1.013 | 0.993, 1.032 | <0.001 |  |
|  | MR-Egger | 0.965 | 0.939, 0.992 | <0.001 | <0.001 |
| T2DM | IVW | 0.962 | 0.829, 1.116 | 0.001 |  |
|  | MR-Egger | 0.878 | 0.670, 1.150 | <0.001 | 0.433 |
| CKD | IVW | 1.128 | 0.983, 1.295 | <0.001 |  |
|  | MR-Egger | 1.017 | 0.790, 1.310 | <0.001 | 0.346 |
| Hyperlipidaemia | IVW | 1.000 | 0.999, 1.001 | 0.078 |  |
|  | MR-Egger | 1.001 | 0.999, 1.003 | 0.081 | 0.337 |

Abbreviations: BMI, body mass index; CI, confidence interval; CKD, chronic kidney disease; DBP, diastolic blood pressure; eGFR, estimated glomerular filtration rate; HDL-C, high-density lipoprotein cholesterol; IVW, inverse variance weighted; LDL-C, low-density lipoprotein cholesterol; OR, odds ratio; SBP, systolic blood pressure; T2DM, type 2 diabetes mellitus.

# Supplementary Table 4. Causal associations of urate with LDL-C and T2DM estimated by the contamination-mixture method based on statistically driven approach

| Outcome | Beta coefficient | 95% CI |
| --- | --- | --- |
| LDL-C | 0.024 | -0.038, -0.020  -0.016, 0.054 |
| Outcome | OR | 95% CI |
| T2DM | 1.027 | 0.845, 1.225  1.292, 1.779 |

Abbreviations: CI, confidence interval; LDL-C, low-density lipoprotein cholesterol; OR, odds ratio; T2DM, type 2 diabetes mellitus.

# Supplementary Table 5. Associations between selected SNPs and urate based on biologically driven approach

| SNP | Chr. | Gene | Effect allele | Other allele | SNP-urate association | | | R^2^ | F-statistic |
| --- | --- | --- | --- | --- | --- | --- | --- | --- | --- |
|  |  |  |  |  | Beta coefficient | SE | P-value |  |  |
| rs10009618 | 4 | *ABCG2* | T | C | -0.089 | 0.006 | 1.98E-51 | 0.00240 | 252.58 |
| rs2231142 | 4 | *ABCG2* | T | G | 0.220 | 0.009 | 4.44E-116 | 0.00528 | 584.47 |
| rs4693935 | 4 | *ABCG2* | G | A | 0.053 | 0.006 | 1.08E-15 | 0.00067 | 70.77 |
| rs10516194 | 4 | *SLC2A9* | C | T | -0.470 | 0.025 | 1.25E-73 | 0.00378 | 353.44 |
| rs11722228 | 4 | *SLC2A9* | T | C | 0.210 | 0.006 | 1.00E-200 | 0.01262 | 1406.25 |
| rs12644047 | 4 | *SLC2A9* | A | G | -0.160 | 0.007 | 2.64E-102 | 0.00486 | 537.70 |
| rs13128385 | 4 | *SLC2A9* | G | C | 0.200 | 0.026 | 1.24E-13 | 0.00064 | 59.17 |
| rs16891971 | 4 | *SLC2A9* | A | C | -0.170 | 0.020 | 1.48E-15 | 0.00066 | 72.25 |
| rs9291642 | 4 | *SLC2A9* | T | C | 0.360 | 0.008 | 1.00E-200 | 0.01999 | 2243.77 |
| rs10498730 | 6 | *SLC17A1* | G | A | -0.084 | 0.012 | 1.30E-10 | 0.00045 | 49.00 |
| rs1165151 | 6 | *SLC17A1* | G | T | 0.092 | 0.005 | 4.52E-60 | 0.00263 | 290.26 |
| rs1359231 | 6 | *SLC17A1* | T | G | 0.073 | 0.008 | 1.52E-17 | 0.00074 | 81.22 |
| rs9467606 | 6 | *SLC17A1* | G | A | 0.074 | 0.010 | 1.84E-13 | 0.00054 | 59.42 |
| rs2078267 | 11 | *SLC22A11* | T | C | -0.078 | 0.006 | 8.73E-36 | 0.00178 | 174.78 |
| rs893006 | 11 | *SLC22A12* | A | C | -0.063 | 0.006 | 1.05E-24 | 0.00107 | 117.98 |

Abbreviations: Chr., chromosome; SE, standard error; SNP, single nucleotide polymorphism.

# Supplementary Table 6. Causal associations of urate with HDL-C estimated by the contamination mixture method at different values of standard deviation parameter (*ψ)* based on biologically driven approach

| Outcome | Standard deviation parameter (*ψ)* | Beta coefficient | 95% CI |
| --- | --- | --- | --- |
| HDL-C | 0.08 | -0.048 | -0.102, -0.014 |
|  | 0.10 | -0.044 | -0.078, -0.015 |
|  | 0.13 | -0.044 | -0.070, -0.015 |
|  | 0.16^a^ | -0.040 | -0.066, -0.014 |
|  | 0.19 | -0.040 | -0.064, -0.015 |
|  | 0.23 | -0.040 | -0.062, -0.015 |
|  | 0.27 | -0.040 | -0.062, -0.015 |
|  | 0.33 | -0.037 | -0.062, -0.014 |

^a^Initial value of the standard deviation of the distribution of invalid estimands (*ψ)*, corresponding to 1.5 times the standard deviation of the ratio estimates.

Abbreviations: CI, confidence interval; HDL-C, high-density lipoprotein cholesterol.

# Supplementary Table 7. Heterogeneity and pleiotropy test results using genetic instrumental variables based on biologically driven approach

| Continuous outcome | Method | Beta | 95% CI | P-value | |
| --- | --- | --- | --- | --- | --- |
|  |  |  |  | Cochran’s Q test | Pleiotropy test |
| BMI | IVW | -0.019 | -0.047, 0.008 | 0.001 |  |
|  | MR-Egger | 0.013 | -0.033, 0.060 | 0.004 | 0.118 |
| SBP | IVW | 0.004 | -0.017, 0.024 | <0.001 |  |
|  | MR-Egger | -0.005 | -0.042, 0.033 | <0.001 | 0.614 |
| DBP | IVW | 0.005 | -0.020, 0.029 | <0.001 |  |
|  | MR-Egger | 0.003 | -0.043, 0.048 | <0.001 | 0.916 |
| Fasting glucose | IVW | 0.007 | -0.008, 0.022 | 0.359 |  |
|  | MR-Egger | 0.008 | -0.019, 0.036 | 0.288 | 0.904 |
| eGFR | IVW | -0.003 | -0.010, 0.003 | 0.004 |  |
|  | MR-Egger | -0.008 | -0.019, 0.004 | 0.004 | 0.379 |
| LDL-C | IVW | 0.011 | -0.016, 0.039 | 0.096 |  |
|  | MR-Egger | 0.032 | -0.017, 0.081 | 0.102 | 0.344 |
| HDL-C | IVW | -0.038 | -0.064, -0.011 | 0.073 |  |
|  | MR-Egger | -0.057 | -0.104, -0.01 | 0.077 | 0.357 |
| Triglycerides | IVW | 0.018 | -0.004, 0.039 | 0.323 |  |
|  | MR-Egger | 0.021 | -0.019, 0.061 | 0.258 | 0.836 |
| Total cholesterol | IVW | 0.008 | -0.016, 0.032 | 0.255 |  |
|  | MR-Egger | 0.018 | -0.027, 0.062 | 0.212 | 0.622 |
| Binary outcome | Method | OR | 95% CI | P-value | |
|  |  |  |  | Cochran’s Q test | Pleiotropy test |
| Hypertension | IVW | 1.001 | 0.991, 1.012 | <0.001 |  |
|  | MR-Egger | 1.003 | 0.983, 1.023 | <0.001 | 0.881 |
| T2DM | IVW | 0.968 | 0.895, 1.047 | 0.985 |  |
|  | MR-Egger | 1.049 | 0.913, 1.206 | 0.998 | 0.193 |
| CKD | IVW | 1.029 | 0.937, 1.131 | 0.077 |  |
|  | MR-Egger | 1.023 | 0.853, 1.226 | 0.052 | 0.933 |
| Hyperlipidaemia | IVW | 1.000 | 0.999, 1.001 | 0.570 |  |
|  | MR-Egger | 1.000 | 0.998, 1.001 | 0.518 | 0.524 |

Abbreviations: BMI, body mass index; CI, confidence interval; CKD, chronic kidney disease; DBP, diastolic blood pressure; eGFR, estimated glomerular filtration rate; HDL-C, high-density lipoprotein cholesterol; IVW, inverse variance weighted; LDL-C, low-density lipoprotein cholesterol; OR, odds ratio; SBP, systolic blood pressure; T2DM, type 2 diabetes mellitus.

# Supplementary Table 8. Causal associations of urate with hypertension from the contamination mixture method based on biologically driven approach

| Outcome | Method | OR | 95% CI |
| --- | --- | --- | --- |
| Hypertension | Contamination mixture | 1.008 | 0.987, 0.994  1.000, 1.015 |

Abbreviations: CI, confidence interval; OR, odds ratio.

# Supplementary Table 9. Causal associations of HDL-C with urate: bidirectional MR

| Pathway | Method | No. of SNPs | Beta coefficient | 95% CI | | P-value |
| --- | --- | --- | --- | --- | --- | --- |
|  |  |  |  | Lower | Upper |  |
| HDL-C → urate | IVW | 85 | -0.115 | -0.180 | -0.032 | 0.001 |
|  | MR-Egger | 85 | -0.004 | -0.112 | -0.053 | 0.943 |
|  | Weighted median | 85 | -0.073 | -0.124 | -0.025 | 0.006 |
|  | Weighted mode | 85 | -0.068 | -0.114 | -0.023 | 0.005 |
|  | MR-PRESSO | 76 | -0.112 | -0.157 | -0.022 | <0.001 |
|  | Contamination Mixture | 85 | -0.066 | -0.133 | -0.032 | 0.001 |

Abbreviations: CI, confidence interval; HDL-C, high-density lipoprotein cholesterol; IVW, inverse variance weighted; MR, Mendelian randomization; No., number; SNP, single nucleotide polymorphism.

# Supplementary Table 10. Causal associations of urate with CVD risk factors estimated by MR-Lasso and MR-RAPS based on statistically driven approach

| Outcome | Method | Beta coefficient | 95% CI | | P-value |
| --- | --- | --- | --- | --- | --- |
|  |  |  | Lower | Upper |  |
| BMI | MR-Lasso | -0.031 | -0.053 | -0.009 | 0.006 |
|  | MR-RAPS | -0.036 | -0.056 | -0.016 | <0.001 |
| SBP | MR-Lasso | 0.046 | 0.024 | 0.068 | <0.001 |
|  | MR-RAPS | 0.028 | 0.017 | 0.040 | <0.001 |
| DBP | MR-Lasso | 0.010 | -0.010 | 0.030 | 0.341 |
|  | MR-RAPS | 0.040 | 0.029 | 0.052 | <0.001 |
| Fasting glucose | MR-Lasso | 0.005 | -0.019 | 0.029 | 0.687 |
|  | MR-RAPS | -0.013 | -0.030 | 0.005 | 0.150 |
| eGFR | MR-Lasso | -0.004 | -0.013 | 0.005 | 0.419 |
|  | MR-RAPS | -0.010 | -0.015 | -0.006 | <0.001 |
| LDL-C | MR-Lasso | 0.014 | -0.019 | 0.047 | 0.400 |
|  | MR-RAPS | 0.024 | -0.001 | 0.05 | 0.064 |
| HDL-C | MR-Lasso | -0.051 | -0.087 | -0.015 | 0.005 |
|  | MR-RAPS | -0.108 | -0.132 | -0.084 | <0.001 |
| Triglycerides | MR-Lasso | 0.072 | 0.039 | 0.105 | <0.001 |
|  | MR-RAPS | 0.320 | 0.297 | 0.343 | <0.001 |
| Total cholesterol | MR-Lasso | 0.026 | -0.013 | 0.064 | 0.189 |
|  | MR-RAPS | 0.055 | 0.031 | 0.08 | <0.001 |
| Outcome | Method | OR | 95% CI | | P-value |
|  |  |  | Lower | Upper |  |
| Hypertension | MR-Lasso | 1.023 | 1.015 | 1.033 | <0.001 |
|  | MR-RAPS | 1.014 | 1.009 | 1.019 | <0.001 |
| T2DM | MR-Lasso | 0.988 | 0.870 | 1.121 | 0.849 |
|  | MR-RAPS | 0.961 | 0.868 | 1.065 | 0.450 |
| CKD | MR-Lasso | 1.088 | 0.974 | 1.213 | 0.135 |
|  | MR-RAPS | 1.131 | 1.038 | 1.232 | 0.005 |
| Hyperlipidaemia | MR-Lasso | 1.000 | 0.999 | 1.002 | 0.377 |
|  | MR-RAPS | 1.000 | 0.999 | 1.001 | 0.698 |

Abbreviations: BMI, body mass index; CI, confidence interval; CKD, chronic kidney disease; CVD, cardiovascular disease; DBP, diastolic blood pressure; eGFR, estimated glomerular filtration rate; HDL-C, high-density lipoprotein cholesterol; LDL-C, low-density lipoprotein cholesterol; OR, odds ratio; SBP, systolic blood pressure; T2DM, type 2 diabetes mellitus.

# Supplementary Table 11. Causal associations of urate with CVD risk factors estimated by MR-Lasso and MR-RAPS based on biologically driven approach

| Outcome | Method | Beta coefficient | 95% CI | | P-value |
| --- | --- | --- | --- | --- | --- |
|  |  |  | Lower | Upper |  |
| BMI | MR-Lasso | -0.014 | -0.035 | 0.008 | 0.215 |
|  | MR-RAPS | -0.020 | -0.036 | -0.003 | 0.023 |
| SBP | MR-Lasso | 0.007 | -0.007 | 0.020 | 0.315 |
|  | MR-RAPS | 0.004 | -0.006 | 0.013 | 0.440 |
| DBP | MR-Lasso | 0.004 | -0.006 | 0.014 | 0.434 |
|  | MR-RAPS | 0.005 | -0.005 | 0.014 | 0.314 |
| Fasting glucose | MR-Lasso | 0.007 | -0.008 | 0.022 | 0.356 |
|  | MR-RAPS | 0.007 | -0.007 | 0.021 | 0.334 |
| eGFR | MR-Lasso | -0.004 | -0.008 | 0.000 | 0.050 |
|  | MR-RAPS | -0.003 | -0.007 | 0.001 | 0.142 |
| LDL-C | MR-Lasso | 0.011 | -0.016 | 0.039 | 0.418 |
|  | MR-RAPS | 0.012 | -0.011 | 0.034 | 0.314 |
| HDL-C | MR-Lasso | -0.038 | -0.064 | -0.011 | 0.005 |
|  | MR-RAPS | -0.038 | -0.059 | -0.017 | <0.001 |
| Triglycerides | MR-Lasso | 0.018 | -0.004 | 0.039 | 0.109 |
|  | MR-RAPS | 0.018 | -0.003 | 0.038 | 0.087 |
| Total cholesterol | MR-Lasso | 0.004 | -0.021 | 0.030 | 0.740 |
|  | MR-RAPS | 0.008 | -0.014 | 0.030 | 0.463 |
| Outcome | Method | OR | 95% CI | | P-value |
|  |  |  | Lower | Upper |  |
| Hypertension | MR-Lasso | 1.000 | 0.992 | 1.008 | 0.998 |
|  | MR-RAPS | 1.002 | 0.997 | 1.006 | 0.468 |
| T2DM | MR-Lasso | 0.969 | 0.895 | 1.047 | 0.419 |
|  | MR-RAPS | 0.968 | 0.895 | 1.047 | 0.420 |
| CKD | MR-Lasso | 1.029 | 0.937 | 1.131 | 0.545 |
|  | MR-RAPS | 1.030 | 0.957 | 1.108 | 0.435 |
| Hyperlipidaemia | MR-Lasso | 1.000 | 0.999 | 1.001 | 0.877 |
|  | MR-RAPS | 1.000 | 0.999 | 1.001 | 0.877 |

Abbreviations: BMI, body mass index; CI, confidence interval; CKD, chronic kidney disease; CVD, cardiovascular disease; DBP, diastolic blood pressure; eGFR, estimated glomerular filtration rate; HDL-C, high-density lipoprotein cholesterol; LDL-C, low-density lipoprotein cholesterol; OR, odds ratio; SBP, systolic blood pressure; T2DM, type 2 diabetes mellitus.

# Supplementary Figure 1. Leave-one-out plots of associations between urate and cardiovascular risk factors based on statistically driven approach.

## Body mass index (BMI)


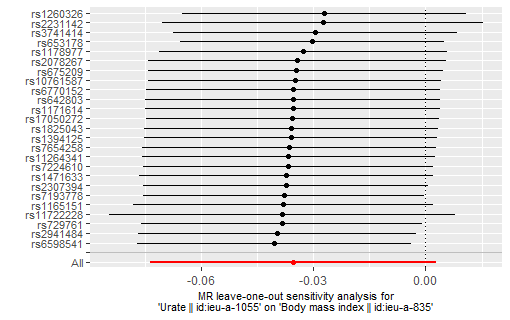


## Systolic blood pressure (SBP)


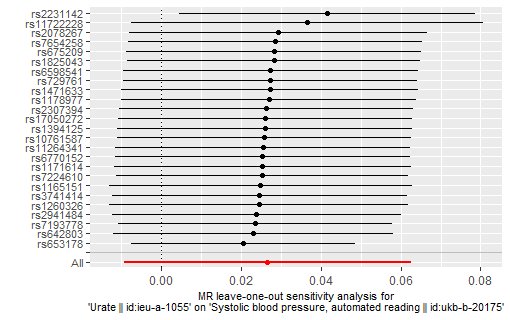


## Diastolic blood pressure (DBP)


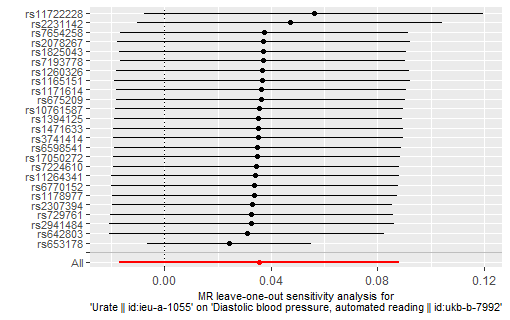


## Hypertension


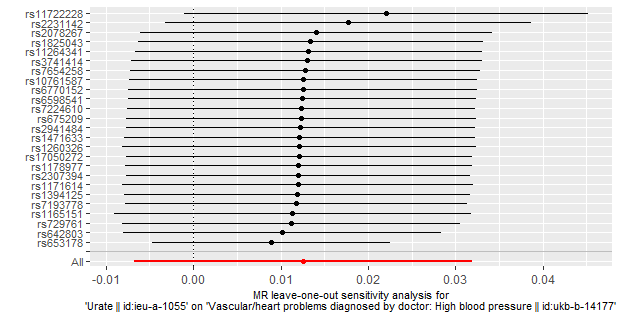


## Fasting glucose


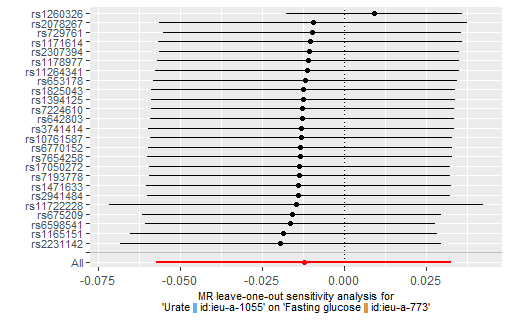


## Type 2 diabetes mellitus (T2DM)


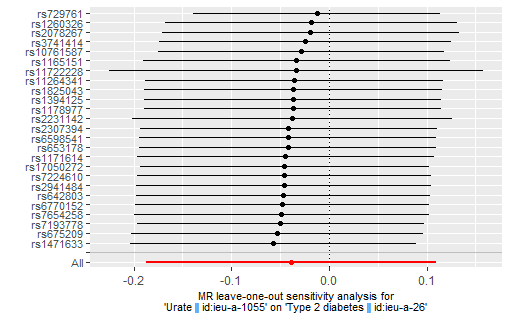


## Estimated glomerular filtration rate (eGFR)


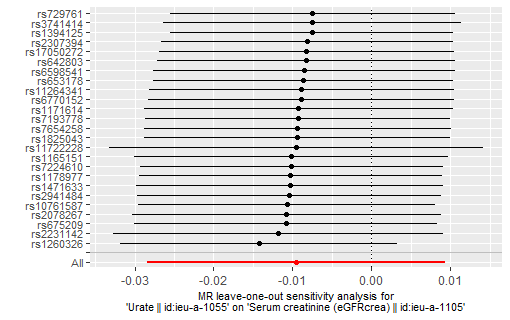


## Chronic kidney disease (CKD)


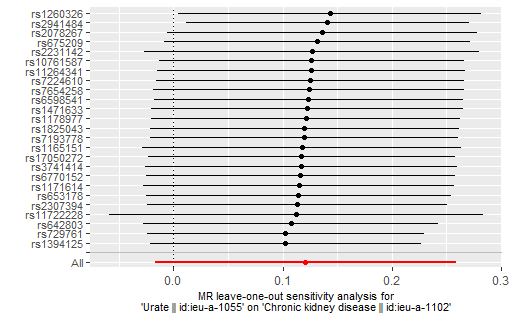


## Low-density lipoprotein cholesterol (LDL-C)


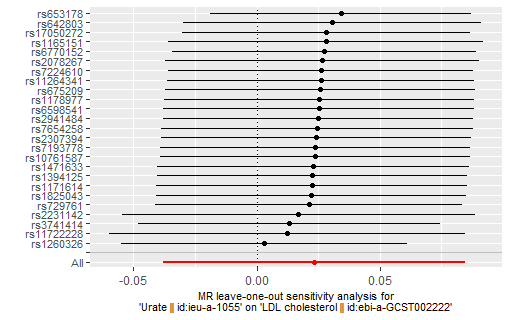


## High-density lipoprotein cholesterol (HDL-C)


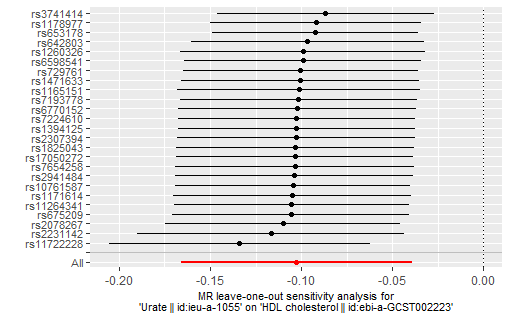


## Triglycerides


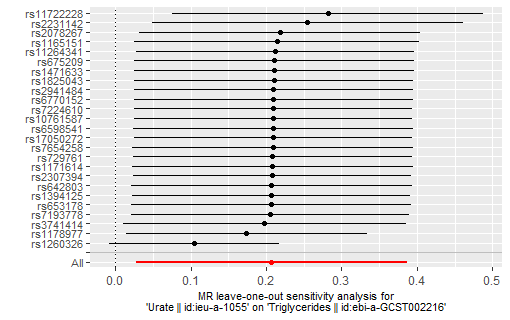


## Total cholesterol


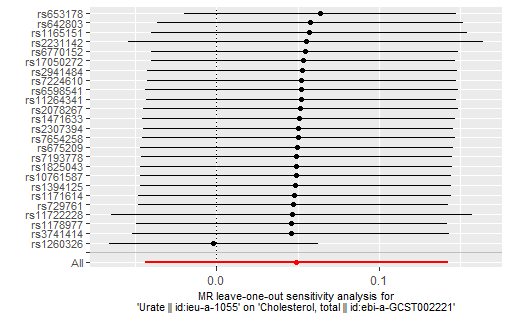


## Hyperlipidaemia


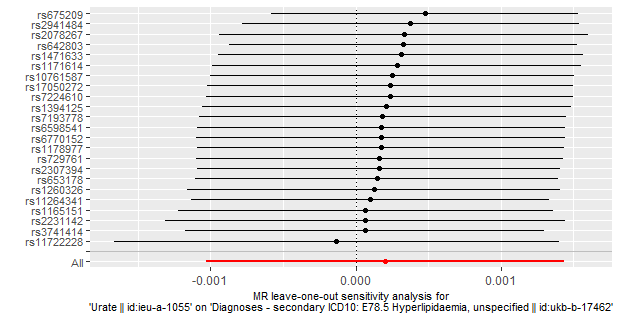


# Supplementary Figure 2. Scatter plots of associations between urate and BMI


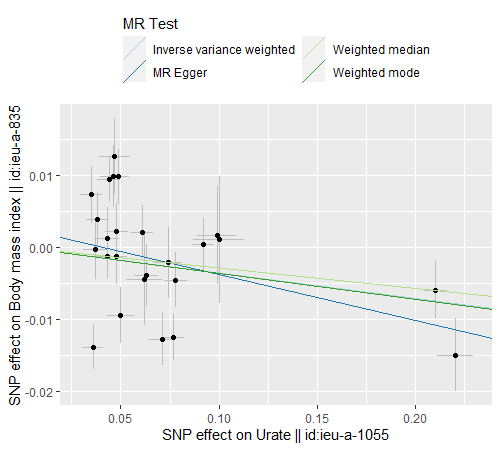


# Supplementary Figure 3. Scatter plots of associations between urate and SBP


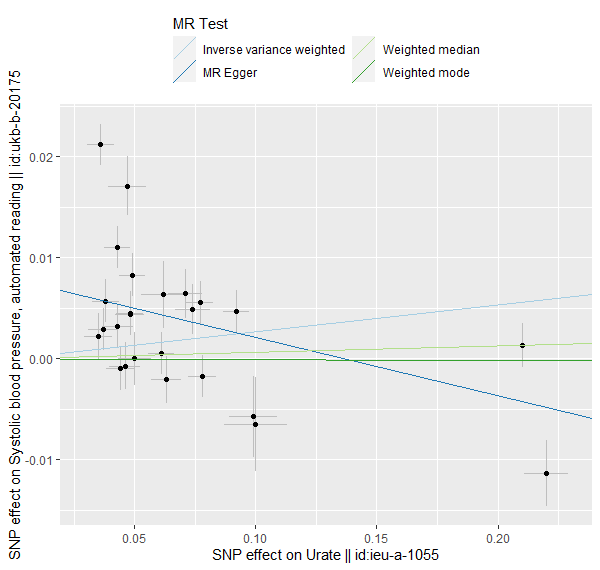


# Supplementary Figure 4. Scatter plots of associations between urate and CKD


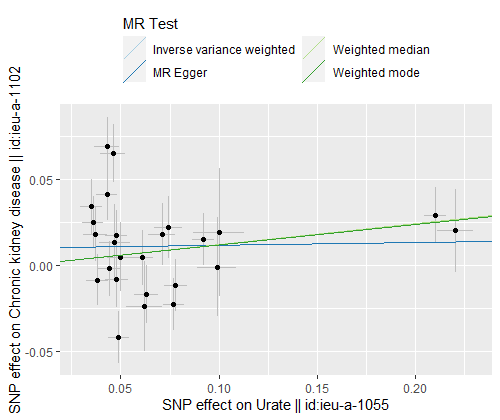


# Supplementary Figure 5. Scatter plots of associations between urate and triglycerides


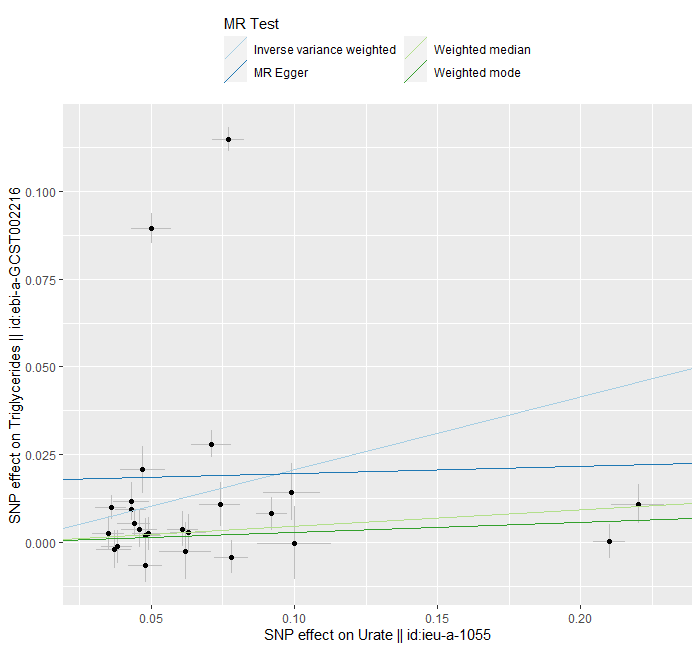


# Supplementary Figure 6. Leave-one-out plots of associations between urate and cardiovascular risk factors based on biologically driven approach

## Body mass index (BMI)


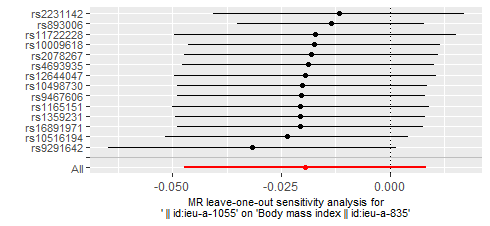


## Systolic blood pressure (SBP)


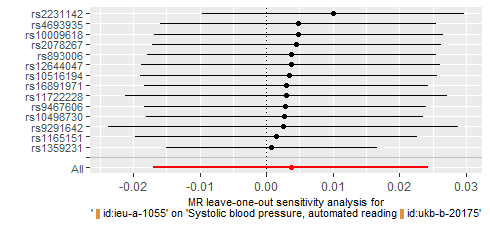


## Diastolic blood pressure (DBP)


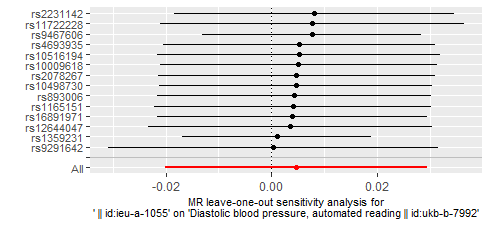


## Hypertension


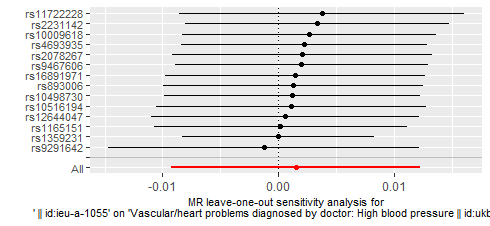


## Fasting glucose


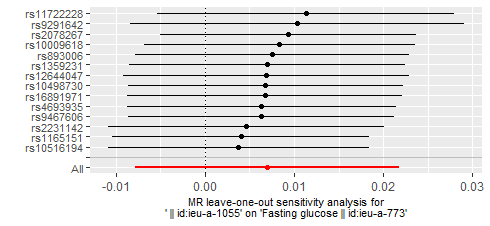


## Type 2 diabetes mellitus (T2DM)


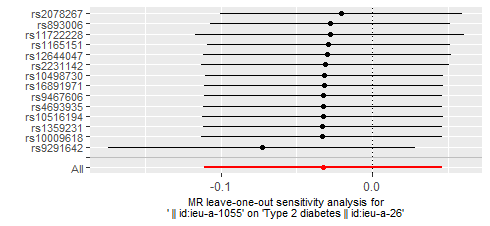


## Estimated glomerular filtration rate (eGFR)


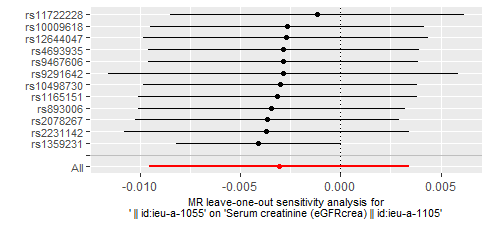


## Chronic kidney disease (CKD)


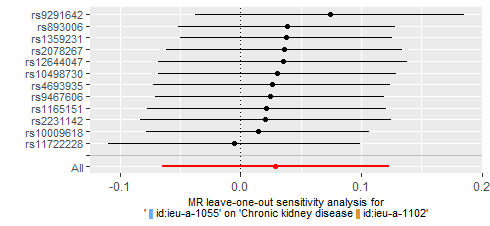


## Low-density lipoprotein cholesterol (LDL-C)


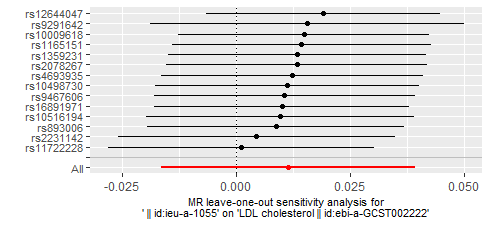


## High-density lipoprotein cholesterol (HDL-C)


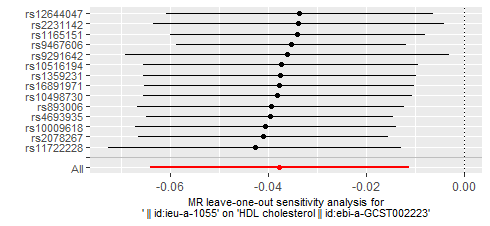


## Triglycerides


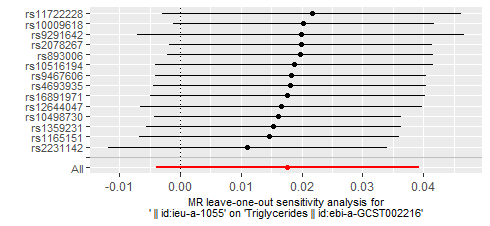


## Total cholesterol


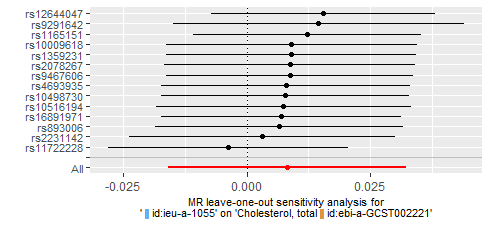


## Hyperlipidaemia


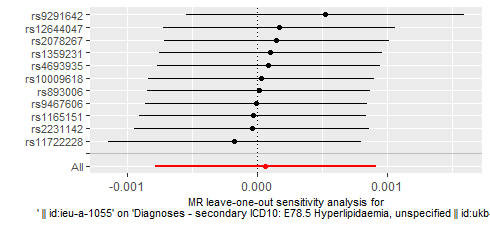


# References

1. Allen N. E., Sudlow C., Peakman T., Collins R. (2014). UK biobank data: come and get it. *Sci Transl Med.* 6, 224ed224. doi: 10.1126/scitranslmed.3008601

2. Collins R. (2012). What makes UK Biobank special? *Lancet.* 379, 1173-1174. doi: 10.1016/s0140-6736(12)60404-8

3. Burgess S., Davey Smith G., Davies N., Dudbridge F., Gill D., Glymour M., et al. (2020). Guidelines for performing Mendelian randomization investigations [version 2; peer review: 2 approved]. *Wellcome Open Research.* 4, doi: 10.12688/wellcomeopenres.15555.2

4. Burgess S., Butterworth A., Thompson S. G. (2013). Mendelian randomization analysis with multiple genetic variants using summarized data. *Genet Epidemiol.* 37, 658-665. doi: 10.1002/gepi.21758

5. Bowden J., Davey Smith G., Burgess S. (2015). Mendelian randomization with invalid instruments: effect estimation and bias detection through Egger regression. *Int J Epidemiol.* 44, 512-525. doi: 10.1093/ije/dyv080

6. Bowden J., Davey Smith G., Haycock P. C., Burgess S. (2016). Consistent Estimation in Mendelian Randomization with Some Invalid Instruments Using a Weighted Median Estimator. *Genet Epidemiol.* 40, 304-314. doi: 10.1002/gepi.21965

7. Hartwig F. P., Davey Smith G., Bowden J. (2017). Robust inference in summary data Mendelian randomization via the zero modal pleiotropy assumption. *Int J Epidemiol.* 46, 1985-1998. doi: 10.1093/ije/dyx102

8. Verbanck M., Chen C. Y., Neale B., Do R. (2018). Detection of widespread horizontal pleiotropy in causal relationships inferred from Mendelian randomization between complex traits and diseases. *Nat Genet.* 50, 693-698. doi: 10.1038/s41588-018-0099-7

9. Burgess S., Foley C. N., Allara E., Staley J. R., Howson J. M. M. (2020). A robust and efficient method for Mendelian randomization with hundreds of genetic variants. *Nature communications.* 11, 376. doi: 10.1038/s41467-019-14156-4

10. Efstathiadou A., Gill D., McGrane F., Quinn T., Dawson J. (2019). Genetically Determined Uric Acid and the Risk of Cardiovascular and Neurovascular Diseases: A Mendelian Randomization Study of Outcomes Investigated in Randomized Trials. *Journal of the American Heart Association.* 8, e012738. doi: 10.1161/jaha.119.012738

11. Allara E., Morani G., Carter P., Gkatzionis A., Zuber V., Foley C. N., et al. (2019). Genetic Determinants of Lipids and Cardiovascular Disease Outcomes: A Wide-Angled Mendelian Randomization Investigation. *Circulation Genomic and precision medicine.* 12, e002711. doi: 10.1161/circgen.119.002711

12. Kottgen A., Albrecht E., Teumer A., Vitart V., Krumsiek J., Hundertmark C., et al. (2013). Genome-wide association analyses identify 18 new loci associated with serum urate concentrations. *Nat Genet.* 45, 145-154. doi: 10.1038/ng.2500

13. Yu T. Y., Jee J. H., Bae J. C., Jin S.-M., Baek J.-H., Lee M.-K., et al. (2016). Serum uric acid: A strong and independent predictor of metabolic syndrome after adjusting for body composition. *Metabolism.* 65, 432-440. doi: <https://doi.org/10.1016/j.metabol.2015.11.003>

14. Kim I. Y., Han K. D., Kim D. H., Eun Y., Cha H. S., Koh E. M., et al. (2019). Women with Metabolic Syndrome and General Obesity Are at a Higher Risk for Significant Hyperuricemia Compared to Men. *Journal of clinical medicine.* 8, doi: 10.3390/jcm8060837

15. Grayson P. C., Kim S. Y., LaValley M., Choi H. K. (2011). Hyperuricemia and incident hypertension: a systematic review and meta-analysis. *Arthritis Care Res (Hoboken).* 63, 102-110. doi: 10.1002/acr.20344

16. Weiner D. E., Tighiouart H., Elsayed E. F., Griffith J. L., Salem D. N., Levey A. S. (2008). Uric acid and incident kidney disease in the community. *J Am Soc Nephrol.* 19, 1204-1211. doi: 10.1681/ASN.2007101075

17. Xu Y. L., Xu K. F., Bai J. L., Liu Y., Yu R. B., Liu C. L., et al. (2016). Elevation of serum uric acid and incidence of type 2 diabetes: A systematic review and meta-analysis. *Chronic diseases and translational medicine.* 2, 81-91. doi: 10.1016/j.cdtm.2016.09.003

18. Chen S., Yang H., Chen Y., Wang J., Xu L., Miao M., et al. (2020). Association between serum uric acid levels and dyslipidemia in Chinese adults: A cross-sectional study and further meta-analysis. *Medicine (Baltimore).* 99, e19088. doi: 10.1097/md.0000000000019088
